# Systematic Review and Meta-Analysis of Renal Biomarkers for Cardiovascular Prognosis: A Look Under the Hood∗

**DOI:** 10.1016/j.jacadv.2023.100764

**Published:** 2024-01-04

**Authors:** Christopher R. deFilippi, Yashika Parashar, Aya Awwad

**Affiliations:** a Harvard Medical School, Boston, Massachusetts, USA; b Inova Schar Heart and Vascular, Falls Church, Virginia, USA

**Keywords:** biomarkers, cardiovascular outcomes, kidney, meta-analysis

**Figure:**
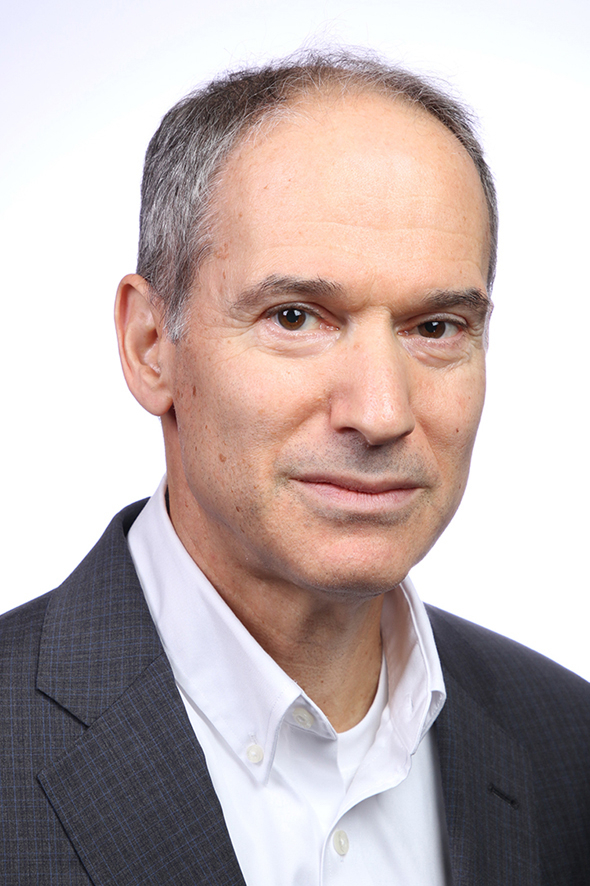

Over the past couple of decades, there has been a veritable tsunami of biomarker publications in cardiovascular disease, and for good reason, because these proteins can be measured in the circulation or urine with high precision at relatively low cost providing mechanistic insights and independent prognostic information in both longitudinal observational cohorts and randomized clinical trials.^1^ However, to all but the most dedicated biomarker enthusiasts, the information is overwhelming, complete with paradoxical results, and ultimately leads to a nihilism to assimilate new biomarkers into practice or even consider new indications for well-established cardiac biomarkers such as cardiac troponins and natriuretic peptide assays. Narrative reviews can be an important step to qualitatively describe biomarkers and their associations with outcomes of interest, but it is the methodological rigor of a systematic review and meta-analysis as high-level evidence, compiling the current knowledge in a specific field, that has the potential to substantiate the conclusions of individual studies into more compelling scientific evidence. The paper by Kumar et al^2^ in this issue of JACC Advances takes a rigorous approach to evaluating renal biomarkers representing glomerular filtration and acute kidney injury with their prognostic associations with mortality, a composite outcome of heart failure hospitalization and mortality, or worsening renal failure. When reviewing a meta-analysis, there can be a tendency to look at the forest plots, or in this case, a nice central figure, and accept the provided point estimates and 95% CIs for the outcomes of interest, but by design, there is so much more here that can inform a reader about both the bias and study heterogeneity effecting these estimates. Here are points to consider when evaluating a meta-analysis including that by Kumar et al.

The process of a meta-analysis, like any carefully conducted study, starts with specification of the research question and development of a written protocol.^3^^,^^4^ This is followed by a search of the literature to identify relevant studies, screening of these studies, analysis of this evidence, and then synthesis in the finished review.^3^^,^^4^ 1) Defining the search strategy is a critical step, as a lack of comprehensiveness and representativeness can bias the conclusions despite statistical rigor.^5^ A good search strategy includes all the relevant original research articles related to the research question in different databases using search terms that are available to the reader, which these authors provided as a detailed search strategy. 2) Predetermine the criteria for subsequent study selection and data extraction including the type of study designs, populations, and other inclusion and exclusion criteria. Lack of adherence to the protocol during extraction can introduce bias into the effect estimates that may be difficult to recognize. In this analysis, the authors included prospective cohort and case control studies as well as secondary data analyses from randomized controlled trials from 100 studies for 3 biomarkers across 3 outcomes. 3) Primary results are shown with forest plots across multiple outcomes to report a pooled effect estimate, ie, the hazard ratio (HR). Pooling odds ratios (used for case control studies) and HRs in a meta-analysis can be challenging due to inherent differences in study designs and data. The odds ratio may not capture time-to-event data accurately, while HRs are influenced by follow-up duration. This may contribute to bias, which must be addressed to interpret the results. 4) Interpretability can be a challenge across studies reporting biomarkers as continuous measures, various quantiles, and study-specific cut points. The authors, by transforming HRs for categorical and continuous variables to the HR of first vs the third tertile, facilitate interpretation across studies.^6^ This approach assumes log-normal distribution of biomarkers and a log-linear association between exposure and outcomes. While seemingly straightforward, when interpreting studies that report HRs differently (ie, per one log change, one log SD change, and categorical), multiple scaling factors need to be applied to derive an accurate conversion to a tertiles interpretation. Inaccurate application will increase bias. 5) The authors performed a *random-effects* model meta-analysis vs a fixed-effects model to analyze the pooled effect estimates. The *fixed-effect model*, which is now rarely used, assumes that one true effect size underlies all the studies in the meta-analysis, while the *random-effects model* assumes that the true effect could vary from study to study due to the differences (heterogeneity) among studies.^7^ The random-effects model can capture uncertainty resulting from heterogeneity among studies. When there are too few studies to obtain an accurate estimate of the between-study variance, one may consider a fixed-effect model.^7^ 6) The assessment of heterogeneity is a critical step to ensure the robustness of findings in a meta-analysis. Heterogeneity can be assessed using a chi-squared test and I^2^ statistics. A low *P* value in the chi-squared test provides evidence of heterogeneity of intervention effects (variation in effect estimates beyond chance), whereas I^2^ describes the percentage of the variability in effect estimates that is due to heterogeneity rather than sampling error (chance). Thresholds for the interpretation of I^2^ should be interpreted carefully after considering several other factors like magnitude and direction of effects and strength of evidence for heterogeneity. I^2^ <40% might not be important, whereas I^2^ 50% to 90% may represent substantial heterogeneity. In the meta-analysis by Kumar et al, particularly for serum cystatin C and N-Gal, they identified substantial heterogeneity. 7) Heterogeneity can be further evaluated in a meta-analysis by predefined subgroups and meta-regression, which was extensively explored by Kumar et al, considering dichotomized factors such as study population, duration of follow-up and continuous variables such as age, none of which, along with other factors, could explain the heterogeneity between studies expressed by the I^2^ statistic. 8) Lastly, understanding and controlling biases in a meta-analysis can be difficult, though tools exist. Every study has some inherent bias. Risk of bias within studies was explored using the Quality in Prognosis Studies tool, which evaluated the risk of selection bias, attrition bias, measurement bias, bias due to confounding, bias due to statistical analysis and presentation of results.^8^ A meta-analysis may have reporting bias and nonreporting bias too. The most frequently assessed reporting bias in a meta-analysis is publication bias. A publication bias usually refers to the bias that arises from the *publication* or *nonpublication* of research findings, depending on the nature and direction of the results.^9^ The authors used funnel plots to explore publication bias by plotting effect estimate against study size. The standard error of the effect estimate is often chosen as the measure of study size and plotted on the vertical axis^10^ with a reversed scale that places the larger, most powerful studies towards the top. An asymmetrical funnel plot can be a result of publication bias resulting in inflated effects in smaller studies. The authors find several examples of publication bias including one of the most extreme, Cystatin C’s association with the composite outcome leading to inflated effects in smaller studies.

In conclusion, both greater heterogeneity and bias can impact the accuracy of the pooled effect estimates (Figure 1). The authors provide a commendable systematic review and meta-analysis for the challenging topic of cardiovascular and renal prognostic associations with several renal biomarkers. Importantly, they recognize marked heterogeneity across studies and potential for publication bias, though they are unable to identify sources for study heterogeneity. These quantifiable findings shed light on the complexity to date of interpreting existing renal biomarker studies and provide potential insights for what needs to be considered in future prospective study designs to improve interpretation of renal biomarkers in cardiac and renal disease.

**Figure 1:**
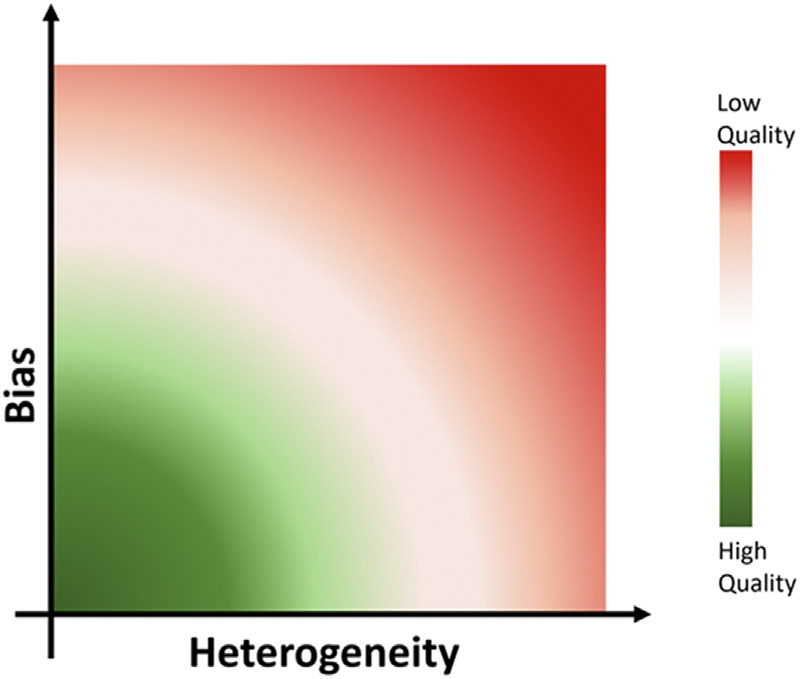
Factors Influencing the Quality of Meta-Analysis Results Heterogeneity is assessed statistically with the Chi-Squared test and/or I^2^. Biases quantitatively assessed include publication bias and study quality. Unmeasured biases including intrinsic biases of the original publications and those introduced by meta-analysis authors during review and data extraction. Both greater heterogeneity and bias can reduce the accuracy of the pooled effect estimates.

## Funding support and author disclosures

The authors have reported that they have no relationships relevant to the contents of this paper to disclose.
